# Principles of Human Physiology

**Published:** 1876-12

**Authors:** 


Principles of Human Physiology. By William B. Carpenter, M.D., F.R.S., F.L.S., Register to the University of London. Edited by Henry Power, M.B., London, F.R.C.S. A new American edition from the eighth revised and enlarged English edition, with notes and additions by Francis G. Smith, Professor of Institnts of Medicine in the University of Pennsylvania, etc. Philadelphia: Henry C. Lea, publisher. 1876.

This popular standard text-book needs no commendation or notice of any kind from us, further than the bare mention of the fact that Dr. Francis Gurney Smith, with notes and additions, edits another American edition of Carpenter's Physiology from the eighth English edition. A more thorough work on physiology could not be found. In this all the new facts discovered by late researches are noticed, and neither student or practitioner should be without this exhaustive treatise on an important elementary branch of medicine.

